# The null distribution of stochastic search gene suggestion: a Bayesian approach to gene mapping

**DOI:** 10.1186/1753-6561-1-s1-s113

**Published:** 2007-12-18

**Authors:** Michael D Swartz, Sanjay Shete

**Affiliations:** 1 Department of Epidemiology, University of Texas M. D. Anderson Cancer Center, P.O. Box 301439, Houston, Texas 77230-1439, USA

## Abstract

Bayesian methods continue to permeate genetic epidemiology investigations of genetic markers associated with or linked to causal genes for complex diseases. The attraction of these methods is an ability to capitalize on Bayesian priors to model additional complexity and information about the disease outside the specific data analyzed. It is well known that the larger the sample size, the more the Bayesian method with uninformative priors can be approximated by its Frequentist analogue. However, what is not known is how much impact the priors have on a Bayesian method when analyzing a null region of the chromosome. Here, we look at the impact of various prior values on stochastic search gene suggestion (SSGS) when analyzing a region of simulated chromosome 6 known to be unassociated with the simulated disease. SSGS is a recently developed Bayesian variable selection method tailored to investigate disease-gene association using case-parent triads. Our findings indicate that the prior probability values do affect false positives, and this study suggests values to calibrate the prior. Also, the sensitivity of the results to the prior probability values depends on two factors: the linkage disequilibrium between the marker loci examined, and whether this dependence is included in the model. In order to assess the null distribution we used the simulated data with the "answers" known.

## Background

With the advancement of computers over the past few decades comes a rise in the application of Bayesian methods and Markov-chain Monte Carlo methods (MCMC) to genetic data (see [1-6] for examples). Many of these MCMC methods capitalize on Bayesian priors to model additional complexity in the problems facing complex disease mapping. Bayesian priors allow prior information about the loci in question to statistically enter the inference regarding the current data set. It is well known that with Bayesian methods, the more data that is collected, the less impact the prior has. Also, using uninformative priors when modeling large data sets generate posteriors that can be approximated by the same asymptotic normal distribution of the corresponding maximum likelihood estimates [7], leading to Bayesian and Frequentist agreement under these conditions.

But how do Bayesian methods behave when there is not a gene to find? Or, what impact does the prior have on inference if, in reality, there are no genes to find? We use simulated data of Problem 3 for the Genetic Analysis Workshop 15 to evaluate the performance of stochastic search gene suggestion (SSGS), a Bayesian method introduced by Swartz et al. [3], in a region of chromosome 6 with no association to disease.

## Methods

SSGS combines hierarchical priors to model alleles within loci with the conditional logistic regression likelihood to model the probability of transmission to a diseased child in case-parent triad data. The full details are given in Swartz et al. [3], and we give a brief overview of the method here.

To review the likelihood, we first define our notation: *D*^+ ^denotes a child is affected; *g *= (*g*_*m*_, *g*_*f*_) denotes the child's genotypes, with each element subscripted with *m *denoting transmitted from the mother, and *f *denoting transmitted from the father; *G*_*p *_denotes the parental genotypes. Then, given the parents *G*_*m *_and *G*_*f*_, the probability of transmitting genotype *g *to the affected child is given by [3,4,8]:

$$P\left(g|D^{+},G_{m},G_{f}\right)=\frac{RR\left[\left(g_{m},g_{f}\right)\right]}{\sum_{g*|G_{m},G_{f}}RR\left[\left(g_{m}^{*},g_{f}^{*}\right)\right]},$$

where g*|*G*_*m*_, *G*_*f *_represents all possible pairings of transmitted and non-transmitted alleles consistent with the parental genotypes and *RR *[(*g*_*m*_, *g*_*f*_)] is a relative risk function defined previously as a conditional logistic regression function [3].

Using generalized transmission-disequilibrium test (GTDT) coding as described by Schaid [8], we must omit a reference allele for model identifiability. Using calculations from Thomas et al. [4], we select the most prevalent allele as our reference allele. Then, using *l *= 1,..., *L *to index the loci, and *a *= 1,..., *A*_*l*_-1 to index the alleles within a locus (and *A*_*l *_denotes the maximum number of alleles at locus *l*), each *β*_*la *_refers to an allele main effect for allele *a *at locus *l*, with the usual interpretation as the log relative risk for transmission to the case. Note that using GTDT coding assumes additive allelic effects [3].

Next, we assume a multivariate normal prior for the main effects, and hierarchical latent indicators to stochastically search through the main effects that impact the probability of transmission [3,9]. We denote the first latent indicator as the vector ***λ ***= (*λ*_1_,..., *λ*_*L*_). Each element of ***λ ***has a Bernoulli distribution with probability *p*_*l*_, where *p*_*l *_is the prior probability that locus *l *is associated with the disease. Similarly, we define a partitioned allele indicator vector, conditional on the loci selected as ***α***|***λ ***= (***α***_1_,..., ***α***_L_) with each partition $\alpha_{l}=\left(\alpha_{l1},...,\alpha_{lA_{l}-1}\right)$ indicating the alleles at locus *l *associated with disease, and each element in ***α***_1 _has a Bernoulli distribution with probability *q*_*la *_as its prior, where *q*_*la *_is the prior probability that locus *l *and allele *a *at locus *l *are associated with the disease. This dual hierarchical prior structure restricts the stochastic search to models that select alleles within loci.

Conditional on the indicators, we can define the prior for main effects. Let *γ*_*la *_= *λ*_*l*_*α*_*la*_. Therefore, ***γ ***indicates the allele main effects when both the locus and allele are indicated. We can define the conditional prior for the coefficients, ***β***|***γ***, as

*π *(***β***|***γ***) = MVN (**0**, **D**_***γ***_**RD**_***γ***_),

where $D_{\gamma }=\text{Diag}\left(k_{l1},...,k_{lA_{l}-1},...,k_{L1},...,k_{LA_{l}-1}\right)$, where each *k*_*la *_is defined as

$$k_{la}=\{\begin{array}{cc} c_{la}\tau_{la} & \text{if }\gamma_{la}=1\\ \tau_{la} & \text{if }\gamma_{la}=0, \end{array}$$

where *c *is large, *τ *is small, and **R **is either an identity matrix or a covariance matrix defined by genetic correlation. (For more details concerning *c *and *τ *and the flexibility of SSGS using these parameters, see Swartz et al. [3] and references therein.)

To define **R**, we start with a blocked matrix, **L**, whose diagonal blocks represent within loci covariance and off-diagonal blocks represent between loci covariance [3]. We model the within-locus covariance by modeling the probability of an allele's presence as a multinomial distribution. The covariance is then defined in the usual way for a multinomial distribution, using the normalized allele frequencies as cell probabilities for the distribution. To model between locus covariance, each element is simply the allele-wise linkage disequilibrium value: $\delta_{i_{a}j_{b}}=p_{i_{a}j_{b}}-p_{i_{a}}p_{j_{b}}$. Once the elements of the blocks are defined, we set **R **= **L**^-1^. More details are given in Swartz et al. [3].

Our posterior distribution is intractable, and therefore, we use MCMC simulations to sample from the posterior distribution. The parameters ***λ ***and ***α ***can be calculated using a Gibbs sampler, while the ***β ***values are updated by locus using a multivariate Metropolis Hastings step [3]. Software to implement SSGS is available at .

We are mainly interested in the marginal posterior of the *γ*_*la *_values given the data. We use the proportion of iterations for each ***γ***_*la *_= 1 to estimate the posterior probability of each allele being associated with transmission to the case. Once the posterior probabilities are calculated, we use the median model decision rule developed by Barbieri and Berger [10]: select genes with posterior probability of inclusion greater than or equal to 0.5.

Recall that conditional logistic regression models the probability of transmission to the affected offspring, and can include other covariates, such as environmental factors in the model. We compare SSGS with the TDT, implemented in TDTEX program in the Statistical Analysis for Genetic Epidemiology release 5.0 (SAGE 5.0) [11]. TDTEX performs McNemar's test of counts of transmitted versus non-transmitted alleles, which is a powerful *χ*^2 ^text of association that cannot include additional covariates.

### Data analysis

We chose to analyze families from Replicates 1 and 2 of Problem 3. Because this method requires case-parent triads, we randomly selected one of the affected sib pairs to be the case. We analyze the first 250 families from both replicates, focusing on six microsatellite markers from simulated chromosome 6: markers 35 to 40. This gave us a total of 59 alleles from Replicate 1 and 57 alleles from Replicate 2. By looking at the answers of the simulation in advance, these markers are far enough away from any of the disease-associated loci to be assumed independent of the disease locus. We analyze these markers under four different prior model specifications. We use the same values for *p*_*l *_and *q*_*la *_as in the sensitivity analysis combined with either using the identity matrix or defining the dependence structure in R as a function of allele frequencies and linkage disequilibrium as in Swartz et al. [3]: 0.5, 0.25, 0.1, 0.01. Additionally, we compare the results from SSGS with standard inference from conditional logistic regression using Stata 8 and the TDT as implemented in TDTEX.

In order to evaluate the method in the presence of a signal, we performed a second analysis of all four methods mentioned above, applied to markers closer to the simulated DR locus, a gene locus involved in increasing risk for the disease. Using the same algorithm as described for the simulated microsatellites [12], we generated three dense microsatellites using the dense single-nucleotide polymorphisms (SNPs) from chromosome 6 at the following locations: 1) 48.40 cM, 2) 49.44 cM, and 3) 51.52 cM. (When constructing the microsatellites, we omitted the SNP located exactly on the DR locus.) From the "answers", we know that dense microsatellite 2 is the closest to the DR locus. Because the simulated signal was so strong, we only used data from Replicate 1 for this analysis.

For these sets of markers, by using the 250 families, not all alleles appeared in our sample, and some alleles had very low frequencies in our sample. Therefore, we considered a minor allele (MA) as any allele with less then a frequency of 4% in our sample, and pooled them to one pseudo-allele. If the pseudo-allele still had less than 4% frequency after pooling the MAs, we then pooled the MA with the least frequent allele with frequency greater than 4% in the data set.

## Results

Using an alpha of 0.05, the conditional logistic regression indicated five alleles from Replicate 1, and seven from Replicate 2. However, after adjusting for multiple testing using Benjamini and Hochberg's FDR method [13], no alleles were significant from either replicate. In Table 1, we report the false-positive rate for SSGS using the identity matrix for **R**. In the first column of Table 1, we report the prior probability of inclusion. Then for each replicate, we report the posterior probability of each locus selected, *p*(*λ*|data) > 0.5, and the number of alleles selected (each allele with *p*(*γ*|data) > 0.5). When we used 0.5 prior probability of inclusion (*p *= *q *= 0.5) we detected 3 of 59 alleles as significant from Replicate 1, and 5 of 57 from Replicate 2, giving an empirical false-positive rate of 7%. When we reduced the prior probability to 0.25, only one allele from Replicate 1 was selected, reducing the rate to 1%, and when we used prior probabilities less than 0.25, no significant loci were detected. Using a covariance structure defined by allele frequencies and linkage disequilibrium for **R**, SSGS did not find any false positives for any prior values of *p *and *q*. TDTEX from SAGE 5.0 indicated Locus 37 with a *p*-value of 0.009 from Replicate 2, which remained significant after implementing the FDR correction. Therefore, conditional logistic regression combined with the FDR correction and SSGS including a dependence structure were the most accurate, while SSGS without including a dependence structure performed acceptably for some values of the prior.

**Table 1:** Loci suggested by SSGS for varying prior probabilities of inclusion using the R = Identity Matrix

	Replicate 1			Replicate 2		
		
Prior (*p *= *q*)	Locus	Posterior locus probability	No. alleles indicated	Locus	Posterior locus probability	No. alleles indicated
0.5	40	0.96	3	40	0.88	3
				38	0.66	2
						
0.25	40	0.85	1	No loci or alleles had greater than 0.5 posterior probability		
						
0.1	No loci or alleles had greater than 0.5 posterior probability					
0.01	No loci or alleles had greater than 0.5 posterior probability					

The methods ranked differently when analyzing the dense microsatellite loci. SSGS without including a dependence structure indicated all alleles at dense microsatellite Locus 2, having posterior probability for all alleles greater than 99% across all prior values of *p *and *q*, yet did not indicate any alleles at dense microsatellite locus 1 or 3. The conditional logistic regression maximum-likelihood estimates also indicated all alleles at dense microsatellite 2 with *p*-values less than 0.001, without indicating any alleles for Loci 1 or 3. When incorporating covariance structure defined as a function of allele frequencies and linkage disequilibrium through **R**, SSGS only indicated one allele at dense microsatellite 2, (allele 14) with posterior probability of 64% when the prior values for *p *and *q *were set equal to 50%. For other smaller values of prior probabilities *p *and *q*, SSGS did not indicate any alleles at any loci. TDTEX indicated both dense microsatellite 2 (*p*-value < 3.3 × 10^-28^) and dense microsatellite 3 (*p*-value < 0.024) to be associated with the simulated disease locus. Here, at some point, all four methods found the marker closest to the disease locus. However, SSGS excluding a dependence structure and the conditional logistic regression gave the strongest evidence for the location of the disease locus, defining the narrowest region; while SSGS including the dependence structure gave the weakest signal, and was also the least robust to different prior probabilities.

The results of this paper differ from the results of our previous work [3]. Previously, we concluded that including the dependence structure in the model was best. However, in these data sets, we see that excluding the dependence structure performed better than including it when looking for a signal. So why did the dependent prior do so well in our previous research? To answer this question we looked at the LD pattern between markers among the dense microsatellites. When looking at the D' values for the dense microsatellites (calculated by ldmax [14]), we found that the LD falls in the range of 0 to 0.24. Meanwhile, the D' values (again calculated by ldmax) for the data for our previous work [3] was much higher with values ranging from 0.25 to 0.52. Therefore, we see a greater improvement gained by including a covariance structure that is a function of allele frequencies and LD when the LD between markers is higher.

## Conclusion

We have revealed important features for SSGS, a new method introduced in Swartz et al. [3]. We found that in null regions, SSGS can be sensitive to the magnitude of prior probability of inclusion, especially when not including dependence structure in the model. To calibrate the prior, this study suggests using a prior probability of inclusion of 0.25 to control for false positives, while not erroneously excluding true loci. In general, we showed that using smaller prior probability of inclusion for loci and alleles reduces false-positive values, particularly in null regions. Most importantly, we found that the usefulness of including genetic dependence between markers in the model depends on the amount of LD between the markers. Therefore, before including the dependence structure in the matrix, it is very important to examine the LD pattern between the marker loci. If the D' values for the markers is less than 0.25, then incorporating a covariance structure based on the LD seems to shrink the signal drastically toward the null and increase the sensitivity to the prior probabilities of inclusion. Therefore, it is better to exclude the dependence structure from the model when using SSGS to minimize false positives in null regions. However, if the LD between the markers is high (greater than 0.25), as in those analyzed in Swartz et al. [3], we see a clear advantage in including dependence structure based on LD into the model.

## Competing interests

The author(s) declare that they have no competing interests.
